# The efficacy of Tuina with herbal ointment for patients with post-stroke depression: study protocol for a randomized controlled trial

**DOI:** 10.1186/s13063-021-05469-1

**Published:** 2021-07-28

**Authors:** Jiming Tao, Lingjun Kong, Min Fang, Qingguang Zhu, Shuaipan Zhang, Sicong Zhang, Jiajia Wu, Chunlei Shan, Ling Feng, Qingjuan Guo, Zhiwei Wu

**Affiliations:** 1 Yueyang Hospital of Integrated Traditional Chinese and Western Medicine, Shanghai University of Traditional Chinese Medicine, 110 Ganhe Road, Hongkou District, Shanghai, 200437 China; 2 Institute of Tuina, Shanghai Institute of Traditional Chinese Medicine, 110 Ganhe Road, Hongkou District, Shanghai, 200437 China; 3 School of Acupuncture-Moxibustion and Tuina, Shanghai University of Traditional Chinese Medicine, 1200 Cailun Road, Pudong New District, Shanghai, 201203 China

**Keywords:** Post-stroke depression, Efficacy, Tuina with herbal ointment, Randomized controlled trial

## Abstract

**Background:**

Post-stroke depression (PSD) is a common complication after stroke which hinders functional recovery and return to social participation of stroke patients. Efficacy of conventional drug therapies for patients with PSD is still uncertain. Therefore, many patients prefer to use complementary and alternative therapies for PSD. Tuina (traditional Chinese manual manipulation) with herbal ointment is an integration of manual therapy, and ointment is an important part of traditional Chinese medicine (TCM) therapy. Preliminary experiments have shown that the Tuina with herbal ointment can improve the mental state of patients with PSD. The purpose of this study is to observe and verify the efficacy of Tuina combined with herbal ointment for patients with post-stroke depression, and to lay a foundation for further research on its mechanism of action.

**Methods/design:**

In this study, a randomized controlled trial will be conducted in parallel, including two intervention groups: Tuina with herbal ointment group and herbal ointment for control group. A total of 84 eligible participants will be randomly assigned to the groups in a 1:1 ratio. All participants will receive conventional antidepressant venlafaxine treatment (75 mg QD), on which they received two different interventions. The interventions for both groups will be carried out 5 times each week for a period of 2 weeks. The primary outcome will be the Hamilton Rating Scale for Depression (HAMD). Secondary outcomes will include transcranial magnetic stimulation (TMS), as well as 36-item Short-Form Health Survey (SF-36) and Treatment Emergent Symptom Scale (TESS). They will be assessed at the baseline, at the end of the intervention (2 weeks), and during the 1 month and 3 months of follow-up by repeated measures analysis of variance. The significance level is 5%. Adverse events will be monitored at each visit to assess safety. All outcomes will be assessed and analyzed by researchers blinded to the treatment allocation. The purpose of this study will focus on observing the efficacy of Tuina with herbal ointment for patients with post-stroke depression, and to explore further the mechanisms of its effects.

**Discussion:**

This study may evaluate clinical application value and safety of Tuina with herbal ointment in PSD patients, which can provide basis for clinical research and mechanism exploration of PSD.

**Trial registration:**

Chinese Clinical Trial Registry ChiCTR2000033887. Registered on 15 June 2020.

**Dissemination:**

The results will be published in peer-reviewed journals and disseminated through the study’s website and conferences.

## Background

Post-stroke depression (PSD), a common complication after stroke, mainly manifests as low mood, sleep disorder, and social withdrawal. It has a negative impact on the daily life activities and cognitive functions of stroke patients [1, 2]. An epidemiological study from (year) showed that the incidence of PSD accounted for about a third of stroke and was higher in the acute phase [3]. PSD severely hampered the functional recovery of stroke patients and caused heavy economic burden to family and society [4]. PSD was even an important predictor of all-cause mortality [5, 6].

There were high estimated differences between antidepressant drugs in clinical trials [7]. No specific class of antidepressants has shown a significant advantage in treating PSD [8]. The PSD clinical medication guidelines lack high-quality evidence-based support, which makes it difficult for clinicians to determine the medication regimen [9]. In addition, first-line antidepressant exposure after intracerebral hemorrhage (ICH) is associated with increasing risk of recurrent hemorrhagic stroke [10]. Therefore, more patients prefer to use complementary and alternative therapies for PSD [11].

Manual therapy, as a complementary and alternative therapy, is gradually recognized by psychiatrists and other mental health practitioners as an effective treatment for mental diseases [12–14]. Manual therapy with specific rhythms and techniques can relieve depression and fatigue [15, 16], such as aromatherapy and Swedish emotional massage therapy, which have been reported to be effective in alleviating depression [17–20]. In China, Tuina (a traditional Chinese manual therapy) combined with herbal ointment is one of traditional Chinese medicine therapies for improving depression, anxiety, etc. [21, 22]. The herbal ointment is composed of essential oils from the Chinese herbs by steam distillation, Vaseline, and glycerine. Some studies reported the effect of the Chinese herbs for post-stroke sleep disorders, cognitive dysfunction, and depression [23]. However, there is no effective evidence of Tuina combined with herbal ointment for PSD.

Study on diffusion tensor imaging (DTI) showed that the PSD patients decreased excitability in brain regions on the ipsilateral side of the stroke lesion, accompanied by cortical and subcortical fiber damage [24]. Disorders of the prefrontal cortex-subcortical neural circuits, cerebellar-hypothalamic loop, and other conduction pathways had been confirmed to be closely related to the pathogenesis of PSD [25]. Systematic reviews showed that Tuina stimulate the C tactile (CT) afferent nerve or associated internal sensory structure to restore the impaired interoceptive functioning [26]. Then certain cortical excitability and connectivity changes occur in the higher centers of the brain through the indirect effects of massage, producing antidepressant effects [27, 28].

Therefore, we hypothesize that the efficacy of Tuina with herbal ointment in the intervention of PSD is different from herbal ointment due to its specific effects on brain neural networks. This study will compare the effect of Tuina with herbal ointment with herbal ointment alone and we assume that Tuina with herbal ointment has a better clinical effect on PSD than herbal ointment alone. Transcranial magnetic stimulation (TMS) will be used to record the activation of cerebral cortex regions [29, 30], which can help us to analyze the changes of brain remodeling in PSD subjects after Tuina with herbal ointment intervention, which is a secondary result we need.

## Methods/design

### Study design

**Fig. 1:**
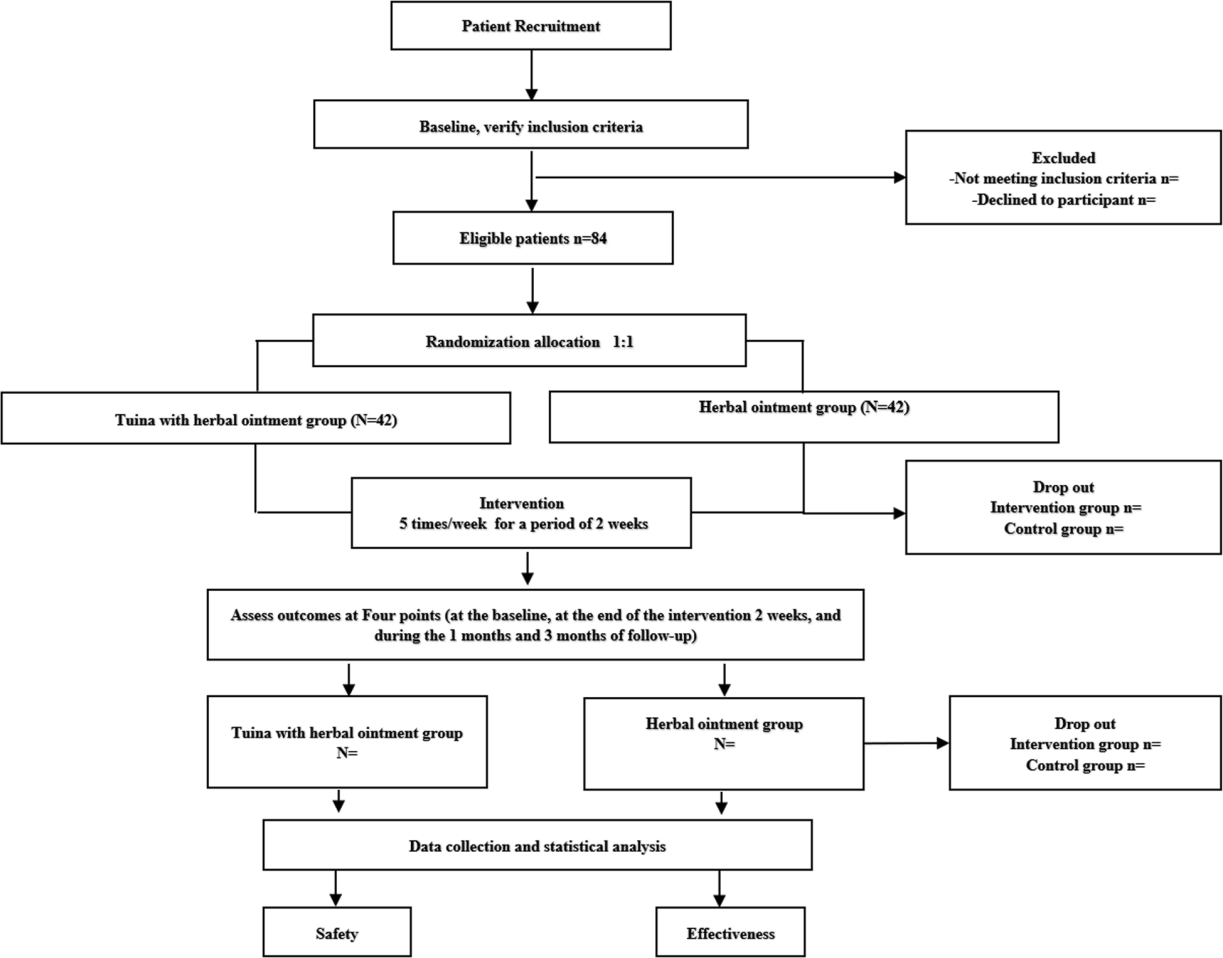
Flow of trial

**Fig. 2:**
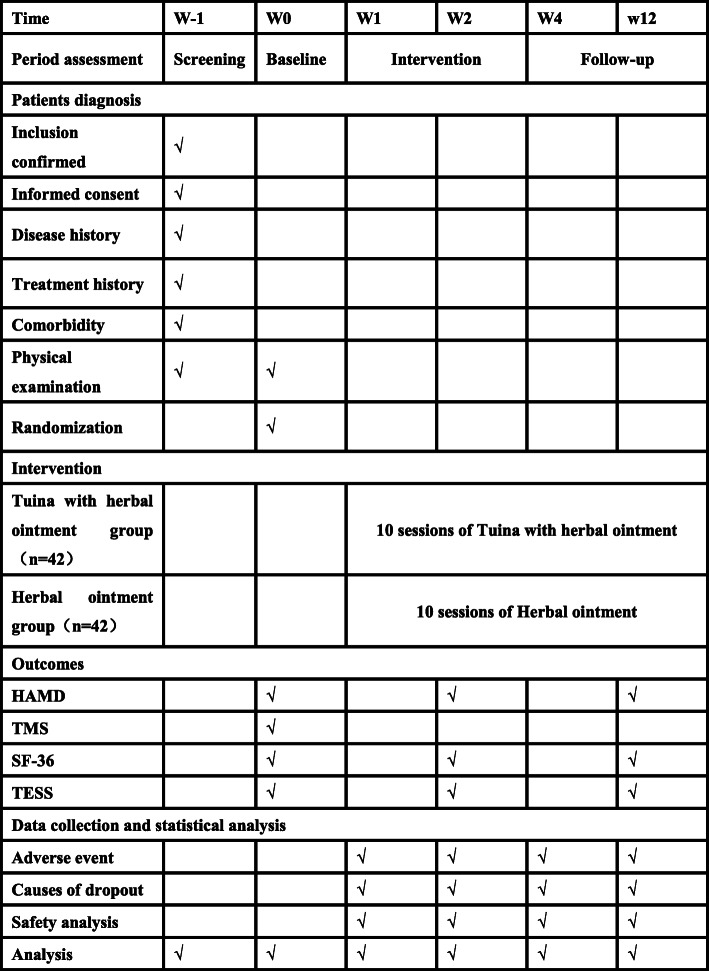
Study schedule data collection showing time points for enrollment and assessment. The informed consent and examination will be conducted after recruitment. After that, we will randomly match PSD patients into two groups, one group will receive Tuina with herbal ointment treatment and the other will receive only herbal ointment. Clinical results will be performed at four time points, including the baseline, at the end of the intervention 2 weeks, and during the 1 month and 3 months of follow-up. During the study, adverse events will be recorded on the case report form. HAMD Hamilton Rating Scale for Depression, TMS Transcranial Magnetic Stimulation, MPQ McGill pain questionnaire, SF-36 36-item Short-Form Health Survey, TESS Treatment Emergent Symptom Scale. W-1 screening before enrollment; W0 baseline assessment; W2 assessment after the 10th treatment, which is in the second week; W4 and W12 are the follow-up period of the 4th and 12th weeks after the first treatment

This study is a two-armed, parallel-design, single-center, assessor- and analyst-blinded, randomized clinical trial conducted in Shanghai, China, which the center is in Yueyang Hospital of Integrated Traditional Chinese and Western Medicine affiliated with Shanghai University of Traditional Chinese Medicine. A total of 84 eligible participants will be randomly allocated at a 1:1 ratio to one of two arms: Tuina with herbal ointment (treatment group) or herbal ointment (control group). This trial protocol has been approved by the Ethics Committee of Yueyang Hospital of Integrated Traditional Chinese and Western Medicine affiliated to Shanghai University of Chinese Medicine (project number:2020-014) and registered in China Clinical Trial Registry (ChiCTR2000033887). Written informed consent will be provided by all patients before the screening. The interventions will be given 5 times per week for 2 weeks. Researchers will be required to assignment to accomplish the outcome assignment and statistical analyses blinded and independently. Figures 1 and 2 illustrate the flow chart and design schedule of the trial in detail.

### Recruitment

Patients will be recruited at the outpatient and inpatient from Rehabilitation departments of Yueyang Hospital of Integrated Traditional Chinese and Western Medicine affiliated with Shanghai University of Traditional Chinese Medicine. Participants will be identified according to the Chinese Guidelines for Cerebrovascular Disease Prevention: A Pilot Edition of Stroke Diagnosis and the Chinese Criteria for the Classification of Mental Disorders (CCMD-3) Diagnostic Criteria for Organic Midbrain Depression [31]. Posters, advertisements, and online platforms will also be used to help us recruit patients.

#### Inclusion criteria

(1) The diagnostic criteria of potential subjects refer to the Chinese Guidelines for Cerebrovascular Disease Prevention [31] and the details are as follows: during the same week, there are 3 or more of the following symptoms, showing different changes from the previous physical and mental function, and at least one of them is required to be a manifestation of a depressive mood: (i) almost every day or most of the time every day has reduced speech (do not want to speak); (ii) fatigue and energy loss almost every day; (iii) depressed mood almost every day or most of the day, subjective description, and observation by others (for example, feeling sad, crying easily); (iv) insomnia, waking up early, or too much sleep almost every day; (v) almost every day or most of the time every day feels that one’s ability is reduced; (vi) recurring thoughts of wanting to die or committing suicide or self-harm; (vii) almost every day or most of the day I feel like I can’t get better; and (viii) almost every day, I am more irritated than usual. The occurrence, development, and course of these symptoms are closely related to cerebrovascular diseases and can cause clinically significant pain or cause damage to social, occupational, or other important functions. At the same time, the appearance of these depressive episodes cannot be better explained by other mental illnesses, such as schizophrenia symptom disorder, and the patient has never experienced manic episodes or hypomanic episodes; (2) between 20 and 80 years of age, male or female; (3) a score of 18 or higher on the first 17 items of the Hamilton Depression Scale (HAMD); (4) willingness to participate in the trial and provide written consent; and (5) participants can understand and follow the protocol.

#### Exclusion criteria

(1) The participants are diagnosed with PSD and has a high-risk willingness to commit suicide; (2) with intellectual disability, language disability, or other serious mental disability and cannot participate in the trial; (3) participants have severe adverse reactions to antidepressants; (4) have an uncomfortable reaction to Tuina; (5) acute (within 2 weeks) or progressive stage of stroke with unstable vital signs; (6) people whose daily behavior is restricted and those who cannot carry out daily activities independently, such as prisoners; and (7) allergic and intolerant to herbal ointment.

#### Drop out criteria

Participants may withdraw from the study for any reason. The following conditions of participants should be considered as withdrawal from the study: (1) failure to stick to the trial, (2) participate in other experiments during the trial, (3) intolerable adverse events, and (4) loss to follow-up.

### Randomization

A statistician unrelated to this experiment will use a computer to make a table of random numbers. Then, participants who complete the basic data assessment will be randomly assigned to Tuina with herbal ointment group and herbal ointment group in a ratio of 1:1. The randomly selected sequence will be sealed in the opaque envelope and provided only to the therapist to allocate the participants accordingly. Assessors and data analysts will be unaware of the group assignments.

### Blinding

It is impossible to blind the therapists and the participants. All other assessors and statisticians will turn a blind eye to group allocation.

### Interventions

Participants will receive a total of 10 sessions over a 2-week period, five times a week for 20 min. The therapists have more than 5 years of experience in clinical massage therapy and received systematic Tuina training. They will also be required to undergo uniform manipulation training prior to the trial to ensure that participants receive homogeneous massage treatments. Considering the tolerance of PSD patients and the consistency of the experiment, the therapeutic site in both groups will be in the back. In addition, no other drugs, surgeries, or alternative therapies are allowed to intervene during the trial, except for basic antidepressant venlafaxine treatment. The main components of the herbal ointment used in the two groups for massage therapy are Vaseline and wintergreen oil extracted from plants (approval number: F20050007). This kind of ointment is the most used for Tuina with herbal ointment.

In addition, during the experiment, we will photograph and record the pressure, frequency, therapeutic targets, and other key data of Tuina and will combine them with the subjective and objective evaluation results for effect correlation analysis. Extract the manipulation elements conducive to the clinical recovery of PSD.

### Tuina with herbal ointment group

Traditional Chinese medicine believes that PSD belongs to the disease of emotion, and its pathogenesis is closely related to the qi disorder of Du meridian [32, 33]. The main position of Du meridian from coccyx ends up to follow the spine into the brain. The principle of Tuina with herbal ointment therapy for PSD is to correct the qi disorder of Du meridian. The therapists use fingers pressing and kneading on the acupoints, palms rubbing along the Du meridian in the spine. The specific operation process is as follows.

First, participants lie on the massage bed in a prone or side position. The acupoints of Du meridian which can improve depression on the back will be located and marked as Baihui (GV20), Dazhui (GV14), Zhiyang (GV9), Mingmen (GV4), and Changqiang (GV1) [34–38]. Apply 1 to 3 g of herbal ointment evenly to each acupoints. The therapists use thumb pressing and kneading those acupoints for about 1–2 min. Then, the fingertips of forefinger, middle finger, and ring finger will be operated for 8–10 min on the Changqiang (GV1) to Dazhui (GV14) line with the rubbing method. Finally, the rapid palm friction method will be applied to the spine of the Du meridian [39]. After the completion of each operation, a “+” was drawn at the operating acupuncture point, which will be used as the body surface positioning symbol for the next operation. One week before the beginning of the trial, the therapist will be in TN-II device (measuring instrument for massage technique) for training, the manipulation pressure set is 0.5 kg, and the frequency is 100 ± 10 times/min (it conforms to the norms of the Tuina of traditional Chinese medicine).

### Herbal ointment group

In the control group, only herbal ointment will be used for intervention, but the dosage and location of drug administration will the same as those in the Tuina with herbal ointment group. The application of herbal ointment in the control group is only for the application of Chinese herbal medicine and does not include the techniques of Tuina while the test group is a comprehensive form of Tuina combined with herbal ointment.

### Measurements

The evaluation of the results will be composed of three rating scales and TMS evaluation data reports, which can reflect the changes in depression, treatment response, quality of life, and the cerebral cortex of PSD participants after different interventions. Four time points will be used to assess respectively, which includes screening, baseline (after enrollment), week 2(intervention session), and months 1 and 3 (follow-up session).

### Primary outcomes

The primary outcome measurement of this experiment is according to the changes in the total HAMD score before and after the 2-week intervention and at 1 and 3 months of follow-up. We will use the HAMD-24 version for this study, which has the highest score of 76 and is widely used by clinicians as a scale tool [40]. HAMD-24 includes 24 problem items, including 7 factor structures, such as anxiety/somatization, body mass, cognitive disorder, day and night change, block, sleep disorder, and despair. A total score of 17 or above is often indicative of mild or higher depression. We will assess the HAMD with a repeated longitudinal analysis.

### Secondary outcome measurement

#### Transcranial magnetic stimulation (TMS)

The brain activity induced by the single transcranial magnetic stimulation (sTMS) and pulse transcranial magnetic stimulation (pTMS) can reflect the excitability of the stimulated cortex. Changes in cortical excitability including motor-evoked potential (MEP), resting motor threshold (RMT), central motor conduction time (CMCT), and cortical resting period (CSP) before and after intervention will be detected by TMS.

#### 36-item Short-Form Health Survey (SF-36)

The SF-36 questionnaire is a global measure of health-related quality of life, which can measure eight scales: physical functioning (PF), role physical (RP), bodily pain (BP), general health (GH), vitality (VT), social functioning (SF), role emotional (RE), and mental health (MH) [41]. Eight SF-36 domains together with the second-order factors will be measured for health-related quality of PSD participants’ life.

#### Treatment Emergent Symptom Scale (TESS)

Treatment Emergent Symptom Scale (TESS) was developed by The National Institute of Mental Health (NIMH) in the USA in 1973. It is a safety assessment tool after treatment of psychiatric diseases. We will use it to evaluate participants’ adverse reactions at a time point of 2 weeks, 1 month, and 3 months.

### Safety assessment

No serious adverse events are anticipated as a result of the study and the clinicians will monitor the risk assessment. In case of adverse reactions caused by antidepressant drugs or massage intervention, we will promptly deal with or withdraw the participant from the study. At the same time, we will record the details in the case report form.

### Follow-up

To observe the changes of participants after the intervention, follow-up evaluations will be conducted at 1 and 3 months, during which participants should only be receiving basic antidepressant treatment. Subjects will be asked to return to the hospital at these two time points for all questionnaire evaluations except TMS. Researchers mainly have the following strategies to ensure the compliance of subjects during follow-up. (1) When the subjects are recruited and signed the informed consent form, the researchers will explain to the patients in detail the needs of the observation period of the subjects and establish a good relationship with the subjects to gain more trust. (2) During the trial, the education and guidance of patient disease protection will be strengthened, and the subjects will receive a certain number of financial subsidies. (3) Finally, this research was carried out in Yueyang Hospital of Integrated Traditional Chinese and Western Medicine Affiliated to Shanghai University of Traditional Chinese Medicine which has professional clinical research foundation and can provide a guarantee for compliance.

### Data collecting and monitoring

All participants will be assessed before treatment, 2 weeks after treatment, and at 1 and 3 months of follow-up. The Department of Rehabilitation of Yueyang Hospital of Integrated Traditional Chinese and Western Medicine affiliated to Shanghai University of Chinese Medicine will act as the data coordination center, responsible for collecting case report forms (CRFs) and data transmission and analysis. All paper data will be stored in secure file cabinets, while electronic data will be protected on password-protected computers. Personnel responsible for data transmission and analysis have only access to de-personalized information of participants.

### Interim analysis

When the subject has a serious adverse reaction, we have a stopping guideline, and the last time the discontinued data is retained will be temporarily analyzed by a dedicated person.

### Statistical analyses

Statistical analyses will be performed by statisticians who are blinded to group allocation. SPSS software 22.0 will be used to run the statistical analysis. The intention-to-treat (ITT) principal will be used to analyze the main sample. Two-sided 5% significance level will be adopted, and the corresponding 95% CI will be calculated as far as possible. A *p*-value of less than 0.05 will be set as significance level. Baseline characteristics will be reported as mean standard deviation (SD). Two-sample *T*-test and repeated measures analysis of variance (ANOVA) will be used for mean differences between and within groups. Nonparametric test will be performed by Wilcoxon test. Chi-square test, Fisher’s exact test, or generalized estimation equation will be used to compare dichotomy variable groups. To assess the maintenance of treatment effectiveness, data after intervention and follow-up (i.e., 2 weeks, 1 month, and 3 months after treatment) will be analyzed repeatedly. Additionally, according to the principle of intentional analysis, the treatment of missing values adopts the method of carrying the last observation result to the end point, so that the number of subjects in each group at the end point is consistent with that at the beginning of the trial. This analysis method ensures randomization in principle.

### Sample size calculation

The objective of this clinical trial is to investigate the efficacy of PSD intervention with Tuina with herbal ointment. The rate of the expectation of the efficacy in Tuina with herbal ointment group is 55%. We estimate the sample size based on the previous research of our research group and the corresponding articles and patents will also be published soon. In the preliminary experiment, the effective rate of herbal ointment application for PSD was 32%. Assuming a dropout rate of 20%, 42 participants will be assigned to each group.

### Quality control

Quality control will be carried out throughout the experimental process. Any modification or adjustment of the experimental scheme will be submitted to the ethics committee and the institution directly under the foundation for approval. The revised protocol will be submitted to the clinical registration center in time to apply for revision and complete the revision to inform all interested researchers. Establish a quality control committee composed of 3–5 experts from the medical technology department, Institute of Tuina and Statistical Teaching and Research Office. Training in data management, massage technique, and evaluation to ensure homogeneity of the intervention program prior to the start of the trial.

## Discussion

We are confident that massage can be clinically effective through the emotional power of touch and psychological interaction with PSD patients [42]. Many PSD patients who received Tuina with herbal ointment had a good treatment experience in China [22, 43]. We designed this trial in order to obtain more supporting clinical evidence and establish a more standardized application of Tuina with herbal ointment to treat PSD. We will try our best to ensure the trial equilibrium, objectively evaluate the efficacy of Tuina with herbal ointment for PSD.

In order to innovate the Tuina with herbal ointment technique for PSD scientifically, we try to find the brain neurobiological mechanism of Tuina with herbal ointment in PSD theory “lesions in the brain, first take the Du meridian” which is the basic TCM theory behind the application of Tuina with herbal ointment for PSD; we are attempting to explore its mechanisms of neurobiological effects in the brain [44]; and we try to explore its mechanisms of neurobiological effects in the brain. Tuina with herbal ointment is essentially a form of touch that produces a pleasant somatosensory sensation. It acts on the patient and affects the higher central cortex through touch receptors, dorsal root ganglia, primary sensory neurons in the brain, and finally the upper central cortex [45]. However, according to the literature review, the neurobiological effects in brain caused by different manipulations techniques are obviously different [44, 46]. We will innovatively use TMS to explore the correlation between Tuina with herbal ointment techniques and the response mechanism of cortical excitability.

### Study limitations

The inevitable limitation of this protocol is that it is difficult to control the blind method during the physical intervention. In this study, due to the different forms of physical therapy, it was not possible for participants and therapists to use the blind method. Therefore, we blinded managers and data analysts to control the credibility of questionnaire assessments. The research tool TMS is relatively simple, and the measurement and evaluation of brain regions is relatively limited. In the future, it is planned to use electroencephalography and functional magnetic resonance imaging to observe the changes of neural networks.

## Trial status

Patients are being recruited from July 2020 to end in June 2022. The registration number is ChiCTR200003388. It is the third version of the protocol on 6 July 2021.

## Data Availability

Not applicable.

## Abbreviations

ANOVA: Analysis of variance
CMCT: Central motor conduction time
CSP: Cortical resting period
CRF: Case report form
CT: C tactile
CCMD: Classification of Mental Disorders
DTI: Diffusion tensor imaging
fMRI: Functional magnetic resonance imaging
HAMD: Hamilton Rating Scale for Depression
ICH: Intracerebral hemorrhage
ITT: Intention-to-treat
MEP: Motor-evoked potential
pTMS: Pulse transcranial magnetic stimulation
PSD: Post-stroke depression
RMT: Resting motor threshold
RCT: Randomized controlled trial
sTMS: Single transcranial magnetic stimulation
SF-36: 36-item Short-Form
SD: Standard deviation
TCM: Traditional Chinese medicine
TMS: Transcranial magnetic stimulation
TESS: Treatment Emergent Symptom Scale
